# Nightly biting cycles of malaria vectors in a heterogeneous transmission area of eastern Amazonian Brazil

**DOI:** 10.1186/1475-2875-12-262

**Published:** 2013-07-26

**Authors:** Robert H Zimmerman, L Philip Lounibos, Naoya Nishimura, Allan KR Galardo, Clicia D Galardo, Mercia E Arruda

**Affiliations:** 1 Florida Medical Entomology Laboratory, University of Florida/IFAS, Vero Beach, Florida, USA; 2 Instituto de Pesquisas Científicas e Tecnologicas de Estado do Amapá, Macapá, Amapá, Brazil; 3 Department of Immunology, Centro de Pesquisas Aggeu Magalhães, Fundação Oswaldo Cruz, Recife, Pernambuco, Brazil

**Keywords:** *Anopheles*, Co-efficient of variation, Host-seeking activity, Kurtosis, Skewness

## Abstract

**Background:**

The biting cycle of anopheline mosquitoes is an important component in the transmission of malaria. Inter- and intraspecific biting patterns of anophelines have been investigated using the number of mosquitoes caught over time to compare general tendencies in host-seeking activity and cumulative catch. In this study, all-night biting catch data from 32 consecutive months of collections in three riverine villages were used to compare biting cycles of the five most abundant vector species using common statistics to quantify variability and deviations of nightly catches from a normal distribution.

**Methods:**

Three communities were selected for study. All-night human landing catches of mosquitoes were made each month in the peridomestic environment of four houses (sites) for nine consecutive days from April 2003 to November 2005. Host-seeking activities of the five most abundant species that were previously captured infected with *Plasmodium falciparum*, *Plasmodium malariae* or *Plasmodium vivax*, were analysed and compared by measuring the amount of variation in numbers biting per unit time (co-efficient of variation, V), the degree to which the numbers of individuals per unit time were asymmetrical (skewness = g_1_) and the relative peakedness or flatness of the distribution (kurtosis = g_2_). To analyse variation in V, g_1_, and g_2_ within species and villages, we used mixed model nested ANOVAs (PROC GLM in SAS) with independent variables (sources of variation): year, month (year), night (year X month) and collection site (year X month).

**Results:**

The biting cycles of the most abundant species, *Anopheles darlingi*, had the least pronounced biting peaks, the lowest mean V values, and typically non-significant departures from normality in g_1_ and g_2_. By contrast, the species with the most sharply defined crepuscular biting peaks, *Anopheles marajoara*, *Anopheles nuneztovari* and *Anopheles triannulatus*, showed high to moderate mean V values and, most commonly, significantly positive skewness (g_1_) and kurtosis (g_2_) moments. *Anopheles intermedius* was usually, but not always, crepuscular in host seeking, and showed moderate mean V values and typically positive skewness and kurtosis. Among sites within villages, significant differences in frequencies of departures from normality (g_1_ and g_2_) were detected for *An. marajoara* and *An. darlingi*, suggesting that local environments, such as host availability, may affect the shape of biting pattern curves of these two species.

**Conclusions:**

Analyses of co-efficients of variation, skewness and kurtosis facilitated quantitative comparisons of host-seeking activity patterns that differ among species, sites, villages, and dates. The variable and heterogeneous nightly host-seeking behaviours of the five exophilic vector species contribute to the maintenance of stable malaria transmission in these Amazonian villages. The abundances of *An. darlingi* and *An. marajoara*, their propensities to seek hosts throughout the night, and their ability to adapt host-seeking behaviour to local environments, contribute to their impact as the most important of these vector species.

## Background

The biting cycle of anopheline vectors is an important component in the transmission of malaria. Studies have been conducted to determine their propensity to bite at particular hours of the night, whether or not they bite inside (endophagic) or outside human dwellings (exophagic), and if they rest inside (endophilic) or outside houses (exophilic) [1-4]. Vector control strategies have been designed based on these studies [5-7]. In the Americas, anopheline species may exhibit late afternoon (e g, *Kerteszia spp*), crepuscular and nocturnal biting activity [8]. Biting cycles have also been classified as having unimodal, bimodal and trimodal activity peaks. Most species studied had similar biting cycles throughout their range, but some species have distinct regional differences. In the Amazon Region, the biting periods of *Anopheles darlingi* can be crepuscular, nocturnal or variants of the two [2,3,9-12]. In French Guyana, Pajot [13] found *Anopheles darlingi* to have a trimodal biting cycle. This species has also been found biting during the day [13-15], Zimmerman, personal observation, Labria, Amazonia, Brazil. The biting cycle of *An. darlingi* also varied seasonally in the same village [12,16]. *Anopheles nuneztovari* showed crepuscular host seeking in many areas of the Amazon Region [2,17], but had a nocturnal biting cycle in western Venezuela and Colombia [1,2,18].

Inter- and intraspecific biting patterns of anophelines have been investigated using the number of mosquitoes caught over time to compare general tendencies in host-seeking activity and cumulative catch. In this study, the co-efficient of variation (V) was used to evaluate variability in numbers biting per unit time, and two measures of deviation from a normal distribution to compare biting cycles, (1) skewness, which is a measure of the asymmetry of a population’s probability distribution [19], and (2) kurtosis, which describes the peakedness and tailedness of a probability distribution [20]. These two parameters, often called moment statistics, were used to describe the shape characteristics of host-seeking activity, and to compare similarities and differences in the activity patterns of anopheline species within and between villages. This study was part of a project on vector heterogeneity and malaria in Amazonian Brazil, where at least five species of *Anopheles* transmit human malaria in three riverine communities [21].

## Methods

### Study area

Three communities separated by 1.5 to 7.0 km, along the Matapí River, Amapá State, Brazil, were selected for study; São Raimundo (00°02′ N; 051°15′ W), São João (00°02′ N; 051°14′W) and Santo Antônio (00°05′N; 051°12′W). A complete description of the region is presented by Zimmerman *et al*[22]. The climate is hot and humid (mean relative humidity 85%) with temperatures ranging from 22-32°C. The rainy season extends from January to July (mean rainfall 2,100 mm), and the dry season from August to December (mean rainfall 178 mm). The ecosystem is a mixed flooded forest (*várzea*) - marsh habitat with many small streams (*igarapés*) draining into the Pirativa and the Matapí River systems. The freshwater level rises and falls with the diurnal tides, flooding the forest floor during high tide, particularly during the rainy season. Malaria is endemic in the area, with *Plasmodium falciparum*, *Plasmodium vivax* VK210, *P. vivax* VK247 and *Plasmodium malariae* being detected in five vector species [21].

### Mosquito collections

All-night human landing catches of mosquitoes were made each month in the peridomestic environment within 5–10 m of four houses (sites) for nine consecutive days (three successive days in each village) from April 2003 to November 2005. Collections occurred during the new moon, when moonlight influence on catch was expected to be minimal [23]. Mosquitoes were collected hourly. Different collectors were used during two 6.5-hour periods from 17:30–00:30 and from 00:30–06:30, and they changed sites and time periods each night to reduce bias or fatigue. Mosquitoes were aspirated as they landed on one exposed leg of the collector and placed in screened 0.5 L ice cream cartons modified as cages. Mosquitoes were placed in a Styrofoam® cooler with wet paper towels and transported to the field laboratory every hour until 22:30. In the morning they were killed with ethyl acetate, and identified to species using the key of Consoli and Lourenço de Oliveira [24].

### Ethical considerations

Use of human subjects was approved by the University of Florida Institutional Review Board (437–2002) and the Brazilian National Ethics Commission for Research (CONEP - 1280/2001).

### Data analysis

For nightly catches of at least 50 individuals of each species, three statistics were calculated to compare and evaluate amount of variation in numbers of mosquitoes landing per hour (co-efficient of variation) and departures from normality (skewness and kurtosis).

### Co-efficient of variation (V)

To assess the relative amounts of variation in numbers per unit time of host-seeking females across species, sites, and sampling dates, we calculated for each nightly collection equal to 50 or more females of a species, the co-efficient of variation, which is the standard deviation expressed as a percentage of the mean.

### Skewness (g_1_) and kurtosis (g_2_)

**Figure 1:**
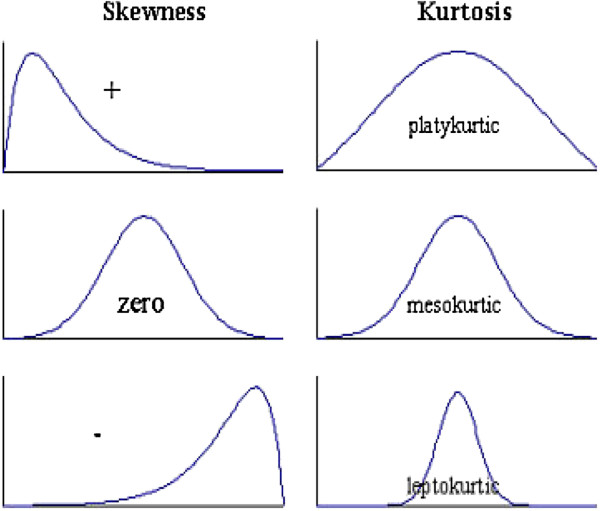
**The distribution patterns - Skewness and Kurtosis.**

Host-seeking activity was analysed for departures from normality by measuring the degree to which the nightly landing catch pattern was asymmetrical (skewness = g_1_) and the relative peakedness or flatness of the distribution (kurtosis = g_2_). A positive skewness indicates the peak is towards the left and the right tail is longer (Figure 1). A negative skewness indicates the peak is towards the right and the left tail is longer. For a normal distribution (mean = median), the skewness co-efficient equals zero.

To assess the degree of kurtosis, we used SAS [25], which subtracts 3 from the kurtosis formula. This correction is done to make the g_2_ value of the normal distribution equal to 0 (mesokurtic); g_2_ values greater than 0 are more peaked (leptokurtic), and those less than 0 are flatter (platykurtic) than a normal distribution (Figure 1). If a mosquito population is leptokurtic, the majority of its host-seeking activity occurs over a shorter time period compared to a mesokurtic or platykurtic distribution. If the mosquito host-seeking activity is platykurtic, its biting activity is dispersed over a greater time period.

### Statistical analyses

To analyse variation in V, g_1_, and g_2_ within species and villages, we used mixed model nested ANOVAs (PROC GLM in SAS) with independent variables (sources of variation): year, month (year), night (year X month) and collection site (year X month). Because the threshold of 50 individuals per species per night was frequently not met, the nested ANOVAs were calculated with unequal sample sizes. For certain species and independent variables, numbers captured were too sparse to run nested ANOVAs.

Mean monthly landing rates for each species in each village were analysed for skewness and kurtosis for each collection site. The frequencies of significantly positive, negative, and non-significant skewness and kurtosis were then analysed for each species in each village by maximum likelihood (ML) categorical analyses of contingency tables using the CATMOD procedure in SAS [25]. If the number of a species collected during the month at a collection site was less than 50, the data were not used for this analysis. Monthly mean human landing catches of the three most abundant species were also analysed for significant effects of year (n = 3), village (n = 3), and species (n = 3) by ML categorical analyses of contingency tables using the CATMOD procedure and ML contrasts to detect significant relationships between independent and dependent variables [25]. To compensate for occasional zero values that were not analysable in PROC CATMOD, 0.001 was added to all zero values in contingency table cells. Significant differences (p < 0.05) in skewness and kurtosis frequency categories among collection sites in each village were evaluated for *An. darlingi* and *Anopheles marajoara*.

## Results

### Mosquitoes collected

Over 32 months of collection, nine anopheline species were identified [21], of which five were abundant enough to be used in this study: *An. darlingi*, *An. marajoara*, *An. nuneztovari*, *Anopheles intermedius*, and *Anopheles triannulatus*. The mean number of mosquitoes (range of the means) collected per night per site varied for each species and in each village [23]. *Anopheles darlingi* was the most abundant species collected (Santo Antônio 874.1 (3.0 – 2324), São João 112.8 (1.5 – 388), São Raimundo 48.3 (0.2 – 214). The second most abundant species was *An. marajoara* (Santo Antônio 358.7 (0.3 – 1230), São João 65.7 (0.0 – 159), São Raimundo 42.5 (0.0 – 289), followed by *An. nuneztovari* (Santo Antônio 70.9 (0.3 – 569), São João 127.7 (4.4 – 780), São Raimundo 42.6 (0.6 – 431). *Anopheles intermedius* (Santo Antônio 48.4 (0.1 – 191), São João 26.8 (0.0 – 370), São Raimundo 9.1 (0.2 – 93) and *An. triannulatus* (Santo Antônio 28.4 (0.0 – 195), São João 8.5 (0.0 – 35), São Raimundo 4.3 (0.8 – 17) were collected the least.

The number of months that mosquitoes were equal or greater than 50 for at least one site in a village was greatest for *An. darlingi* (Santo Antônio = 27, São João = 21, São Raimundo = 21), followed by *An. marajoara* (Santo Antônio = 27, São João = 23, São Raimundo = 17), *An. nuneztovari* (Santo Antônio = 10, São João = 27, São Raimundo = 9), *An. intermedius* (Santo Antônio = 15, São João = 4, São Raimundo = 4) and *An. triannulatus* (Santo Antônio = 9, São João = 3, São Raimundo = 0).

### Host-seeking activity

**Figure 2:**
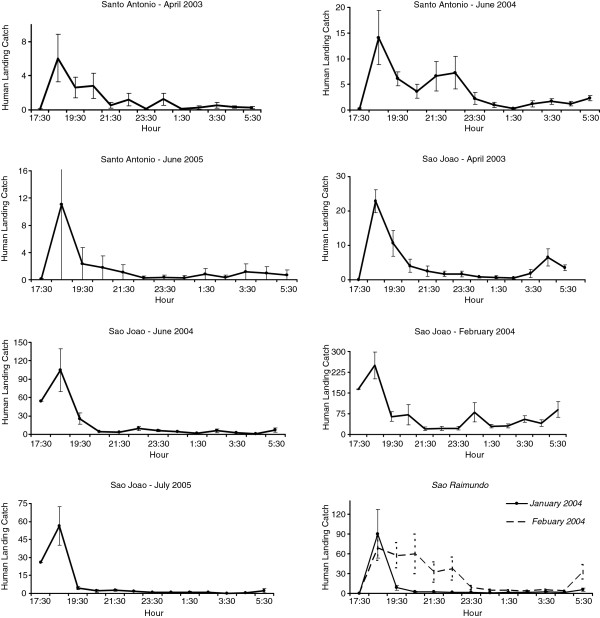
**Mean(±SE)monthly human landing catches for *An*. *nuneztovari*.**

**Figure 3:**
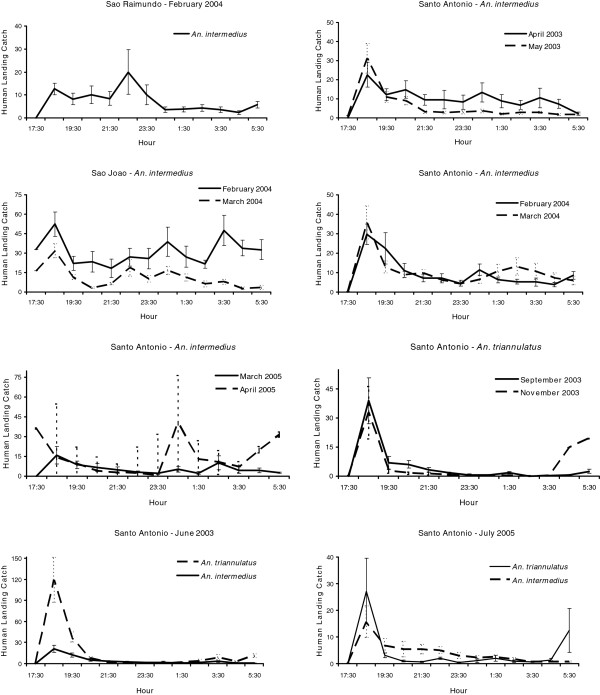
**Mean (±SE) monthly human landing catches for *An*. *intermedius *and *An*. *triannulatus*.**

**Figure 4:**
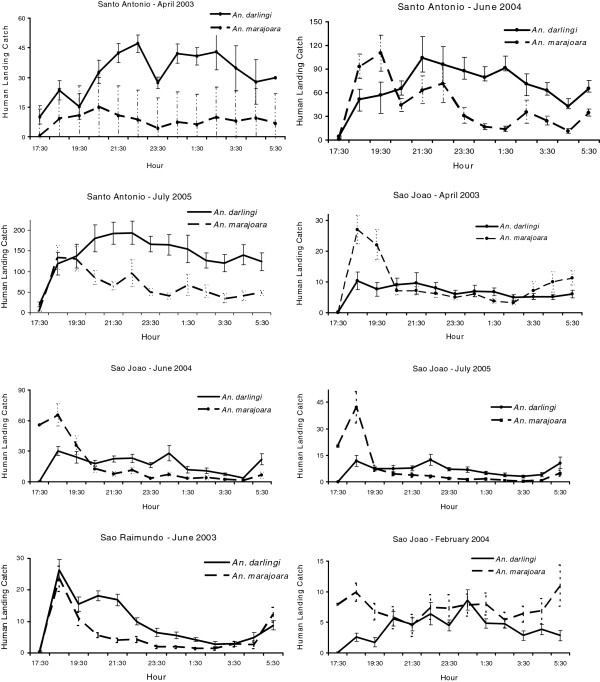
**Mean (±SE) monthly human landing catches for *An*. *darlingi *and *An*. *marajoara*.**

**Figure 5:**
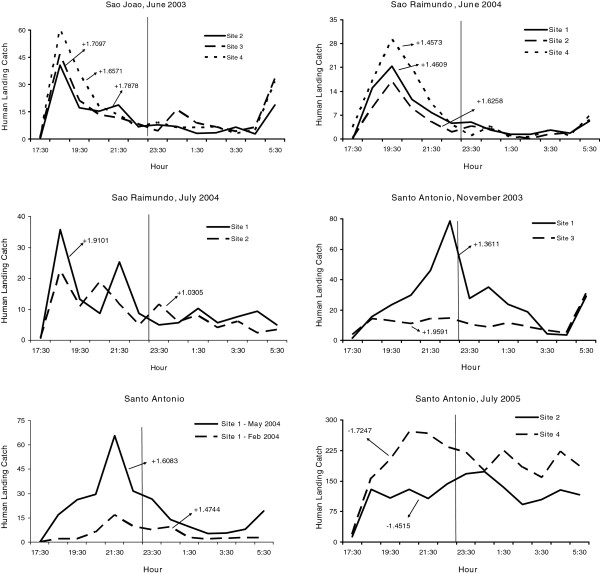
**Mean numbers caught at human landing catches and significant skewness moments for *An*. *darlingi *(P < 0**.**05).**

Variability in biting times was observed among species, villages, and sites. *Anopheles nuneztovari* and *An. triannulatus* showed mostly crepuscular activities with the majority biting from 17:30 to 21:30 (Figure 2 and Figure 3, Additional files 1, 2, 3 and 4). The activity patterns of *An. marajoara* and *An. intermedius* varied; most of their activity peaked between 18:30–19:30. However, during some collections their activity continued throughout the night (Figures 3 and 4, Additional files 1, 2, 3 and 4). *Anopheles darlingi* host-seeking activity occurred from 17:30 until 06:30 (Figure 4, Additional files 1, 2 and 3). Exceptions to these patterns occurred for each species. For example, in São Raimundo and in São João (June 2003), *An. darlingi* was most active between 18:30 and 21:30 (Figures 4 and 5). *Anopheles nuneztovari* had a prolonged activity until just before 24:00 in São Raimundo (Figure 2), and evening and morning peaks in Santo Antônio, March 2005 (Additional file 1).

### Co-efficients of variation (V)

Mean V was highest (i e, showed the greatest intraspecific variation) for the three species of *Nyssorhynchus*, *An. marajoara*, *An. nuneztovari*, and *An. triannulatus*, which typically have crepuscular biting peaks (Table 1, Table 2, Table 3, Table 4, Figure 2, Figure 3, Figure 4). Lower mean Vs were estimated for *An. intermedius* and, especially, *An. darlingi*, whose biting peaks were less frequently crepuscular (Table 1, Table 2, Table 3, Table 4, Figure 3 and Figure 4).

**Table 1:** **Results of nested ANOVA for coefficients of variation** (**V**) **of*Anopheles darlingi *hourly landing rates in three Amazonian villages**

**Model**					**Year**		**Month(year)**		**Night(year x mo.)**		**Site(year X mo.)**	
**Village**^**a**^	**df**	**F**	**R**^**2**^	**meanV**	**df**	**F**	**df**	**F**	**df**	**F**	**df**	**F**
SA	163	1.98***	0.675	69.13	2	10.43***	24	5.52***	52	0.71ns	84	1.45*
SJ	124	3.11***	0.804	88.97	2	0.53ns	20	7.42***	37	2.31***	62	1.58*
SR	79	2.29***	0.767	100.83	2	0.09ns	14	3.36***	23	1.64ns	39	2.56***

^**a**^ SA - Santo Antônio, SJ – São João, SR – São Raimundo; Significant differences *** = P<0.001, * = P<0.05, ns = non-significant.

**Table 2:** **Results of nested ANOVA for co**-**efficients of variation** (**V**) **of*Anopheles marajoara *hourly landing rates in three Amazonian villages**

**Model**					**Year**		**Month(year)**		**Night(year x mo.)**		**Site(year X mo.)**	
**Village**^**a**^	**df**	**F**	**R**^**2**^	**meanV**	**df**	**F**	**df**	**F**	**df**	**F**	**df**	**F**
SA	161	4.39***	0.830	126.04	2	1.06ns	26	12.70***	51	2.83***	81	2.25***
SJ	135	5.26***	0.916	159.59	2	4.81*	22	14.19***	42	3.23***	69	1.89**
SR	79	2.39**	0.820	151.91	2	0.71ns	12	3.75***	23	3.03***	41	1.04ns

^**a**^ SA - Santo Antônio, SJ – São João, SR – São Raimundo; Significant differences *** = P<0.001, ** = P<0.01, * = P<0.05, ns = non-significant.

**Table 3:** **Results of nested ANOVA for coefficients of variation** (**V**) **of*Anopheles nuneztovari *hourly landing rates in three Amazonian villages**

**Model**					**Year**		**Month(year)**		**Night(year x mo.)**		**Site(year X mo.)**	
**Village**^**a**^	**df**	**F**	**R**^**2**^	**meanV**	**df**	**F**	**df**	**F**	**df**	**F**	**df**	**F**
SA	57	5.35***	0.908	163.24	2	1.80ns	11	10.69***	17	2.83**	26	3.08**
SJ	126	3.09***	0.868	236.08	2	7.16**	25	6.98***	38	3.19**	58	1.07ns
SR	42	2.80**	0.831	204.50	1	7.10***	8	7.08***	15	2.72*	17	1.09ns

^**a**^ SA - Santo Antônio, SJ – São João, SR – São Raimundo; Significant differences *** = P<0.001, ** = P<0.01, * = P<0.05, ns = non-significant.

**Table 4:** **Results of nested ANOVA for coefficients of variation** (**V**) **of*Anopheles triannulatus*** (**At**) **and*Anopheles intermedius*** (**Ai**) **hourly landing rates in two Amazonian villages**

**Model**					**Year**		**Month(year)**		**Night(year x mo.)**		**Site(year X mo.)**	
**Village**^**a**^	**df**	**F**	**R**^**2**^	**meanV**	**df**	**F**	**df**	**F**	**df**	**F**	**df**	**F**
SA *(At)*	55	3.26**	0.913	224.08	2	3.77*	14	5.00**	16	3.53**	22	1.47ns
SA *(Ai)*	88	3.97***	0.904	131.21	2	8.02**	15	2.64**	29	3.53**	41	3.46***
SJ *(Ai)*	26	3.27*	0.867	111.54	2	5.96*	7	5.75**	7	2.94*	10	1.23ns

^**a**^ SA - Santo Antônio, SJ – São João; Significant differences *** = P<0.001, ** = P<0.01, * = P<0.05, ns = non-significant.

Nested ANOVA models accounted for significant variation in V of numbers per unit time for all species in all villages (Table 1, Table 2, Table 3, Table 4). The year variable was significant for some species in some villages, with no clear pattern. The nested variable month (year) was significant for all species in all villages, perhaps indicative of seasonal influences on V. Significant variation in V among nights (year X month) was detected for all species in all villages, except for *An. darlingi* in Santo Antônio and São Raimundo (Table 1, Table 2, Table 3, Table 4). Significant variation in V among collection sites (year X month) was detected for *An. darlingi* in all three villages (Table 1). For the other four species, the importance of this variable varied among villages and species (Table 2, Table 3, Table 4).

### Skewness

Nested ANOVA models showed significant variation in skewness for *An. darlingi* only in São Raimundo, not in Santo Antônio or São João, but significant variation in skewness was detected in all three villages for *An. marajoara*. Independent variables that were most commonly significant in these models were month nested in year, and night nested in year X month (Additional file 5).

**Figure 6:**
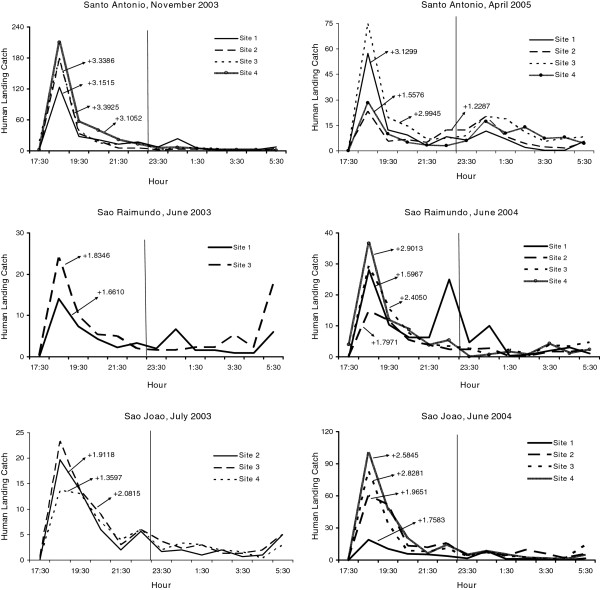
**Mean numbers caught at human landing catches and significant skewness moments for *An*. *marajoara *(P < 0.05).**

**Figure 7:**
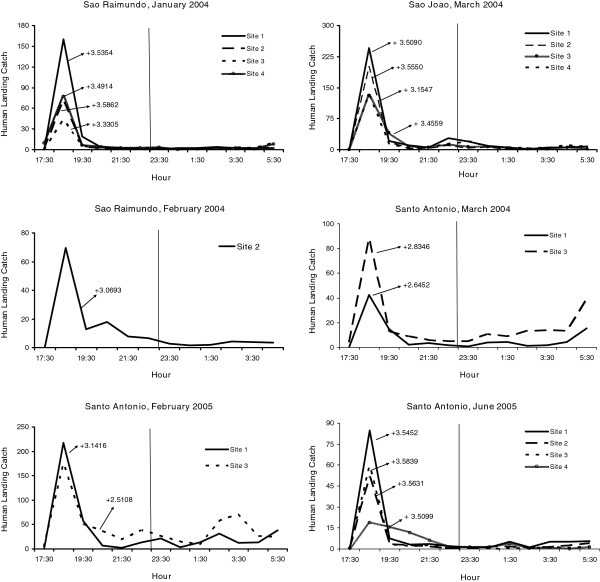
**Mean numbers caught at human landing catches and significant skewness moments for *An*. *nuneztovari *(P < 0.05).**

**Figure 8:**
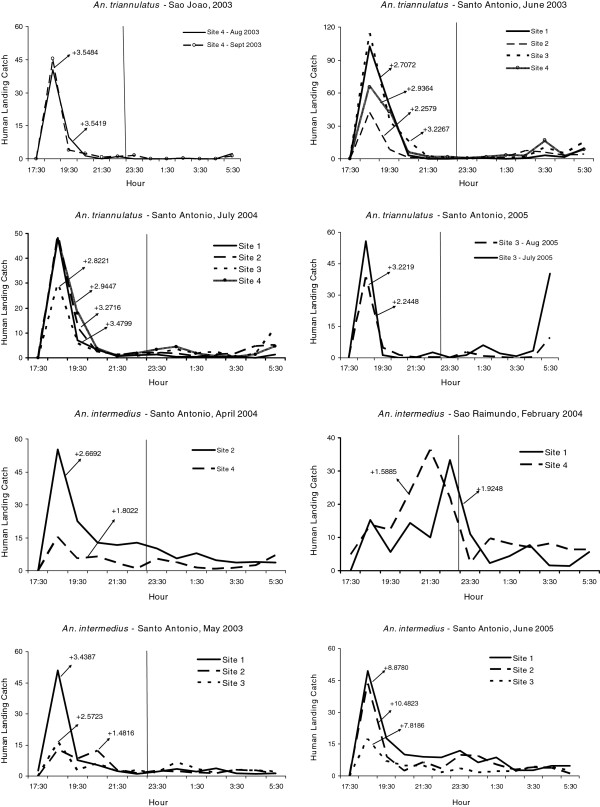
**Mean numbers caught at human landing catches and significant skewness moments for *An*. *intermedius *and *An****. ****triannulatus *(P < 0.05).**

For monthly mean abundances, host-seeking patterns of *An. darlingi* were positively skewed or not significantly different from normal, except for three negatively skewed distributions in Santo Antônio (Figure 5, Table 5). Eighty-three percent of the skewness co-efficients (183/219) were not significantly different from normal while 15.1% (33/219) of the human landing catch activity patterns were positively skewed. Skewness results for *An. darlingi* were in contrast to *An. marajoara*, *An. nuneztovari*, and *An. triannulatus*, in which the significantly positive skewness rates were much higher; 82.3% (177/215), 88.6% (101/114) and 95.6% (21/22), respectively*. Anopheles intermedius* biting activity patterns were mostly skewed significantly in a positive direction (58.9%, 33/56) with 41.1% (23/56) not significantly different from normal (Table 5). Examples of the nightly biting numbers with significant skewness moments (g_1_) are presented in Figures 6, 7, 8.

**Table 5:** **Frequencies of positive and negative skewness** (g_**1**_) **by species in each village**

**Species**	**Village**^**a**^	**Positive**	**Negative**	**NS**^**b**^	**Total**
*An. darlingi*	SA	5	3	94	102
	SJ	14	0	59	73
	SR	14	0	30	44
	Total	33	3	183	219
*An. marajoara*	SA	73	0	24	97
	SJ	65	0	8	73
	SR	39	0	6	45
	Total	177	0	38	215
*An. nuneztovari*	SA	21	0	7	28
	SJ	62	0	3	65
	SR	18	0	3	21
	Total	101	0	13	114
*An. triannulatus*	SA	18	0	1	18
	SJ	3	0	0	3
	SR	0	0	0	0
	Total	21	0	1	22
*An. intermedius*	SA	26	0	14	40
	SJ	3	0	8	11
	SR	4	0	1	5
	Total	33	0	23	56

^**a**^ SA - Santo Antônio, SJ – São João, SR – São Raimundo.

^**b**^ NS - no significant difference from normal at P < 0.05.

The ML ANOVA for skewness frequency categories of the three most abundant species collected showed no significant effects for year, village, species, or interactions. The ML contrasts showed a significant difference in frequencies of skewness categories between *An. darlingi* and *An. nuneztovari* (P = 0.0005), and no significant difference in skewness categories between *An. marajoara* and *An. nuneztovari* (P = 0.4007) or between *An. darlingi* and *An. marajoara* (P = 0.0707) at 5% experiment-wise error rate after Bonferroni corrections (Table 6). ML ANOVAs of skewness frequency categories were not calculated for *An. triannulatus* and *An. intermedius* because of the low number of sites with greater than 50 females of these species (Table 5).

**Table 6:** **Contrasts of maximum likelihood estimates for significant effects of year**, **village**, **and species on skewness for*Anopheles darlingi***, ***Anopheles marajoara*and*Anopheles nuneztovari***

**Contrast**	**DF**	**Chi-Square**	**P-value**
2003 *vs* 2004	2	0.01	0.9929
2003 *vs* 2005	2	0.02	0.9885
2004 *vs* 2005	2	0.00	0.9998
SA *vs* SJ	1	0.00	0.9940
SA *vs* SR	1	0.00	0.9962
SJ *vs* SR	1	0.00	0.9973
*An. darlingi vs An. marajoara*	1	3.27	0.0707
*An. darlingi vs An. nuneztovari*	1	12.17	0.0005^**a**^
*An. marajoara vs An. nuneztovari*	1	0.71	0.4007

^**a**^*P* value is significant at the 5% experiment-wise error rate after Bonferroni correction.

The host-seeking activities of *An. darlingi* and *An. marajoara* were further examined by analysing the effects of collection site on the frequency of different skewness response categories (pos, neg, ns) within each village. The CATMOD procedure showed no significant difference in the frequency of skewness categories of *An. darlingi* by collection site in São Raimundo, but significant intersite differences were detected in São João and Santo Antônio (Table 7). Performing the same analyses for *An. marajoara* revealed intersite differences in skewness response frequencies only in São Raimundo (Table 7).

**Table 7:** **Frequencies of positive and negative skewness** (g_**1**_) **for*Anopheles darlingi*and*Anopheles marajoara*by collection site in each village**

		***An*.*darlingi***				***An*.*marajoara***	
**Village**	**Site**	**Pos**	**Neg**	**NS**^**a**^	**Pos**	**Neg**	**NS**^**a**^
São Raimundo^**b**^	1	4	0	5	10	0	1
	2	2	0	7	10	0	0
	3	4	0	9	10	0	1
	4	4	0	9	9	0	4
São João^**c**^	1	1	0	18	8	0	3
	2	7	0	9	19	0	1
	3	4	0	16	20	0	2
	4	2	0	16	18	0	2
Santo Antônio^**d**^	1	3	0	23	16	0	8
	2	0	1	22	15	0	7
	3	2	0	24	22	0	5
	4	0	2	25	20	0	4

^**a**^ NS - no significant difference from normal at P < 0.05.

^**b**^ For *An. marajoara* in SR, Site 4 frequency distributions were significantly different from all others at 5% experiment-wise error after Bonferroni corrections.

^**c**^ For *An. darlingi* in SJ, frequency distribution in Site 1 was significantly different from Sites 2–4 at 5% experiment-wise error after Bonferroni corrections.

^**d**^ For *An. darlingi* in SA, frequency distributions in Site 1 and Site 3 were significantly different from Sites 2 and 4 at 5% experiment-wise error after Bonferroni corrections.

### Kurtosis

Nested ANOVA models showed significant variation in kurtosis for *An. darlingi* only in São Raimundo, not in Santo Antônio or São João, but significant variation in kurtosis was detected in all three villages for *An. marajoara*. In the significant ANOVA models independent variables that accounted for significant variation were month nested in year, and night nested in year X month (Additional file 5).

**Figure 9:**
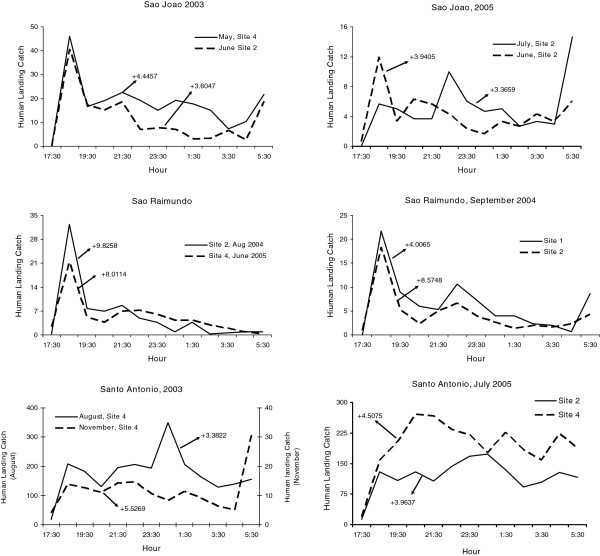
**Mean numbers caught at human landing catches and significant kurtosis moments for *An*. *darlingi *(P < 0.05).**

**Figure 10:**
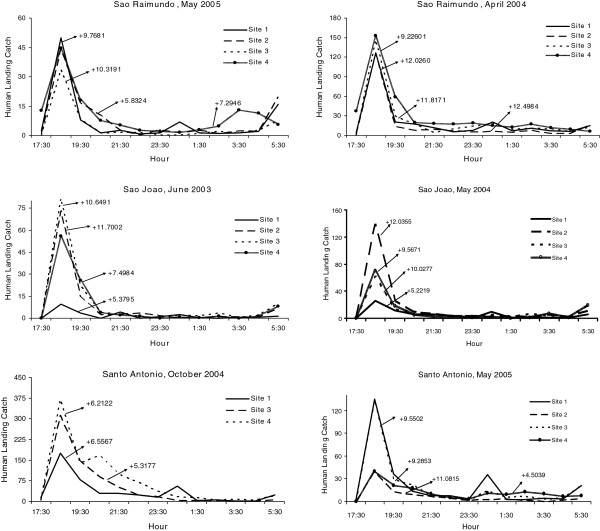
**Mean numbers caught at human landing catches and significant kurtosis moments for *An*. *marajoara *(P < 0.05).**

Kurtosis co-efficients for monthly mean numbers of *An. darlingi* were positive (leptokurtic) or not significantly different from normal (mesokurtic) except for one negative kurtosis in Santo Antônio and one in São João (Table 8). One hundred and ninety-eight monthly collections (90.4%, 198/219) had kurtosis patterns for *An. darlingi* that were not significantly different from normal; while 8.7% (19/219) of the distributions demonstrated significantly positive kurtosis (leptokurtic). Examples of sites that showed significant kurtosis are shown in Figure 9. *Anopheles marajoara* kurtosis co-efficients were significantly positive for 58.1% (125/215) of the collections, e g, Figure 10, and non-significant for 40.9% (88/215) of the distributions (Table 8). Significant negative kurtosis for this species was detected twice; both times at Site 1 in Santo Antônio during August and September 2003.

**Table 8:** **Frequencies of kurtoses** (**g**_**2**_) **with significant deviations from normality by species in each village**

**Species**	**Village**^**a**^	**Positive**	**Negative**	**NS**^**b**^	**Total**
*An. darlingi*	SA	6	1	95	102
	SJ	7	1	65	73
	SR	6	0	38	44
	Total	19	2	198	219
*An. marajoara*	SA	42	2	53	97
	SJ	52	0	21	73
	SR	31	0	14	45
	Total	125	2	88	215
*An. nuneztovari*	SA	17	0	11	28
	SJ	56	0	9	65
	SR	13	0	8	21
	Total	86	0	28	114
*An. triannulatus*	SA	17	0	2	19
	SJ	2	0	1	3
	SR	0	0	0	0
	Total	19	0	3	22
*An. intermedius*	SA	19	0	21	40
	SJ	1	1	9	11
	SR	2	0	3	5
	Total	22	1	33	56

^**a**^ SA - Santo Antônio, SJ – São João, SR – São Raimundo.

^**b**^ NS - no significant difference from normal at P < 0.05.

**Figure 11:**
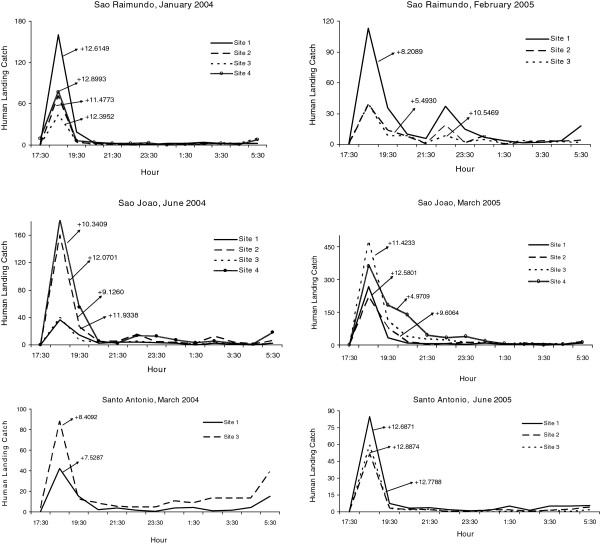
**Mean numbers caught at human landing catches and significant kurtosis moments for *An*. *nuneztovari *(P < 0.05).**

**Figure 12:**
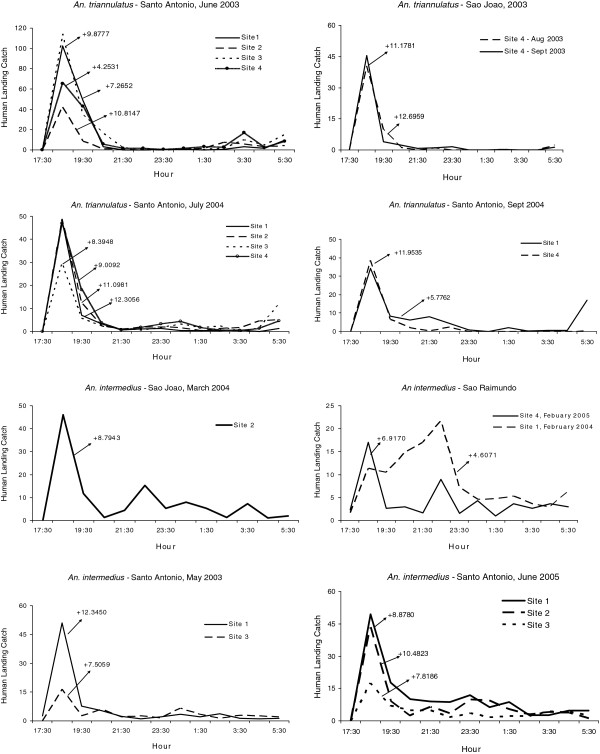
**Mean numbers caught at human landing catches and significant kurtosis moments for *An*. *triannulatus *and *An*. *intermedius *(P < 0.05).**

Significant positive kurtoses for *An. nuneztovari* were detected in 75.4% (86/114) of the collections (e g, Figure 11), and the remainder 24.6% (28/114) were not significantly different from normal (Table 8). No nightly collections of this species showed significant negative kurtosis. *Anopheles triannulatus* kurtosis co-efficients were 86.4% (19/22) significantly positive and 13.6% (3/22) did not differ significantly from normal (Table 8, Figure 12). For *An. intermedius*, significantly positive kurtosis occurred in 39.3% (22/56) of the distributions, and 58.9% (33/56) were not significantly different from normal (Table 8). One site in São João had significant negative kurtosis. Significant kurtoses of *An. intermedius* are presented in Figure 12.

The ML ANOVA for kurtosis for the three major species collected showed no significant effects of year, village, or interactions. The ML contrasts showed a significant difference in kurtosis frequency categories between *An. darlingi* and *An. nuneztovari* and between *An. darlingi* and *An. marajoara* (Table 9). No significant difference in kurtosis frequency categories was detected between *An. marajoara* and *An. nuneztovari* (Table 9).

**Table 9:** **Contrasts of maximum likelihood estimates for significant effects of year**, **village**, **and species on kurtosis for*Anopheles darlingi***, ***Anopheles marajoara*and*Anopheles nuneztovari***

**Contrast**	**DF**	**Chi-Square**	**P-value**
2003 vs 2004	2	0.17	0.9187
2003 vs 2005	2	0.02	0.9900
2004 vs 2005	2	0.82	0.6648
SA vs SJ	2	2.62	0.2695
SA vs SR	2	1.46	0.4808
SJ vs SR	2	0.06	0.9724
*An. darlingi* vs *An. marajoara*	2	74.08	<0.0001^**a**^
*An. darlingi* vs *An. nuneztovari*	2	15.67	0.0004^**a**^
*An. marajoara* vs *An. nuneztovari*	2	1.81	0.4055

^**a**^*P* value is significant at the 5% experiment-wise error rate after Bonferroni correction.

Variability in kurtosis frequency categories of *An. darlingi* and *An. marajoara* was further examined among collection sites for each village. The CATMOD procedure showed significant differences in kurtosis frequency categories for *An. darlingi* by collection site in São João but not in Santo Antônio (Table 10). In São Raimundo, Site 2 was significantly different from the other three sites for both species at 5% experiment-wise error after Bonferroni corrections (Table 10).

**Table 10:** **Frequencies of positive and negative kurtosis (g**_**2**_**) for*Anopheles darlingi*and*Anopheles marajoara*by collection site in each village**

**Village**			***An*.*darlingi***			***An*.*marajoara***	
	**Site**	**Pos**	**Neg**	**NS**^**a**^	**Pos**	**Neg**	**NS**^**a**^
São Raimundo^b^	1	3	0	6	4	0	7
	2	0	0	9	0	0	9
	3	2	0	11	3	0	8
	4	1	0	12	6	0	7
São João^c^	1	0	0	19	5	0	6
	2	5	0	11	4	0	16
	3	1	0	19	5	0	17
	4	1	1	16	7	0	13
Santo Antônio	1	1	1	24	12	2	10
	2	1	0	22	17	0	5
	3	1	0	25	13	0	14
	4	3	0	24	11	0	13

^**a**^ NS - no significant difference from normal at P < 0.05.

^**b**^ For both species, significant differences occurred between Site 2 and Sites 1,3,4 at 5% experiment- wise error after Bonferroni corrections.

^**c**^ For *An. darlingi* significant differences occurred between Site 1 and Sites 2,3,4 at 5% experiment-wise error after Bonferroni corrections.

## Discussion

### General patterns and variability

Previous reports from the Amazon region showed that *An. darlingi* had two main biting periods (crepuscular and nocturnal), with the pattern varying depending on the geographical location of the collection [3,14,26]. In our study area, *An. darlingi* was generally active from 18:30 until 06:30 (Figure 4, Additional files 1, 2 and 3), and its nightly biting numbers per unit time showed the lowest variability (V) among the five studied species (Table 1, Table 2, Table 3, Table 4). Our collections throughout the night also call into question studies from other areas in Amazonian Brazil that ranked importance of malaria vector species based solely on crepuscular collections [16,27].

*Anopheles nuneztovari* and *An. triannulatus* were the two species that showed consistent crepuscular activity, with the vast majority of mosquitoes biting soon after dusk and tapering off rapidly around 20:00 to 21:00. Both species, however, exhibited relatively high co-efficients of variation (V) in numbers per unit time (Table 3 and Table 4).

Very little is known about *An. intermedius* in the Amazon Region. We have previously reported on its local potential as a vector of malaria [21], host preference [22] and seasonality [23]. Human landing catches showed that *An. intermedius* nightly biting patterns varied considerably (Figures 3, 8, 12, Additional file 4), the two villages with sufficient numbers for nested ANOVA analyses showing significant variation in V between years and among months and nights (Table 4).

### Skewness and kurtosis patterns

Skewness and kurtosis patterns of *An. darlingi* were markedly different from those of the other four species. The g_1_ and g_2_ moments for *An. darlingi* were typically non-significant, with few exceptions, which are associated with the predominantly nocturnal (17:30–06:30) activity pattern of this species, rather than the crepuscular pattern typical of *An. marajoara*, *An. nuneztovari* and *An. triannulatus*. The human landing catch profiles of *An. darlingi* did vary among sites and villages. The skewness frequency analysis demonstrated that significant differences occurred between sites in two villages. In São João, Site 1 had significantly lower positive skewness frequencies than Sites 2–4 (Table 7). This difference could have been due to the fact that Site 1 was at the end of the village that ran parallel to the Matapi River, where fewer people lived (one residence) compared to the other sites in São João. The isolation of this site may have caused a delay in biting activity until later in the evening. Site 4 was most similar to Site 1 (11% positive skewness) and was at the other end of the village with two houses near the collection site. Site 3 (20% positive skewness) was closer to the centre of the village and near a corral that had many pigs. Site 2 (43.8% positive skewness) was at the midpoint of the village where many people gathered at dusk. These results suggest that the availability of hosts influences early evening attraction, which probably accounts for the increases in significantly positive skewness. All the sites were equally distant from potential larval habitats of *An. darlingi*.

In contrast, in Santo Antônio the houses were located uphill, perpendicular to the river. Site 1 was along the Matapì River and next to a small stream (*igarapé*), while Site 2 was the first site up the hill from the river, Site 3 was at the back side of the village but very close to a larval habitat, and Site 4 was to the north of the village near a buffalo corral. The position of Sites 1 and 3 near larval habitats may have influenced the skewness patterns, suggesting a relation to the proximity of potential aquatic habitats and resting places. However, the main difference in skewness frequencies was that Site 2 and Site 4 had one and two collecting periods, respectively, with significantly negative skewness (Table 7). Little difference was apparent in percent of positively skewed activity patterns (7.4% to 11.5%). More detailed studies would be needed to examine intrapopulation skewness differences within a village.

Difference in kurtosis frequencies was observed for *An. darlingi* and *An. marajoara* in São Raimundo; all the kurtosis moments for Site 2 were non-significant, while the other sites had some positive kurtoses. As stated before for intra-site skewness comparisons, the differences between Site 2 and the other sites were that more people lived near this site and the community gathered here late into the night. Prolonged human activity around this site may have extended the biting activity, producing a higher percentage of non-significant kurtosis frequencies compared to the other three sites. In São João, all the kurtosis moments for Site 1 were non-significant for *An. darlingi*, while the other sites had some positive kurtosis. Site 1 was at the end of the village with only one residence. The lack of human activity around this site may have shortened its biting activity.

Charlwood [3] reviewed the factors that possibly influence the form of the biting cycle of *An. darlingi*. He suggested that the age of the population, moonphase and distance from the oviposition or mating site may influence the biting pattern of this species. In our study, we observed that the distance from larval habitats may have influenced anopheline activity patterns within a village. Therefore, characteristics of the site of collection need to be considered when examining host-seeking activity patterns of these species. Studies conducted over longer time periods and at various sites would provide better inferences about the factors that influence *An. darlingi*’s activity patterns. This would further clarify the biting behaviour plasticity proposed by several authors [3,16]. Further research on these factors is warranted for all the vector species studied.

The percentage of sites that had significantly positive co-efficients of skewness and kurtosis were high for *An. nuneztovari* and *An. triannulatus*, the positive skewness typically derived from ‘tails’ in nocturnal biting activity after the crepuscular peak, and the leptokurtosis showing that the biting peak is characteristically large in relation to tails (Figures 2 and 3). The crepuscular activity peaks associated with significantly positive skewness were typically captures made in the second hour of nightly collections (Figures 5, 6, 7, 8), suggesting that these deviations from a normal distribution are not caused by inadequate sampling (e g, in pre-dusk hours) of potential biting times.

ML ANOVA comparisons showed no significant difference in the frequencies of skewness or kurtosis categories between *An. nuneztovari* and *An. marajoara* (Table 6, Table 7, Table 8, Table 9). However, *An. marajoara* had a higher percentage of mesokurtic distributions (41.9%) (i e, not significantly different from normal) compared to *An. nuneztovari* (24.6%) (Table 8 and Table 9). This difference is evident by visual inspections of their activity patterns: *An. marajoara* host-seeking activity was more dispersed, peaking between 18:30 and 19:30 with additional activity throughout the night, while *An. nuneztovari* activity occurred mainly between 17:30 and 21:30 (Figures 2 and 4, Additional files 1, 2 and 3). Differences among sites within villages in skewness and kurtosis frequency categories (Table 7 and Table 10) suggest that *An. marajoara* may be similar to *An. darlingi* in its capacity to alter activity patterns in response to environmental cues, such as host availability.

The skewness and kurtosis co-efficients for *An. intermedius* were usually significantly positive or not significantly different from normal, with one exception for kurtosis in São João (Table 5 and Table 8). Insufficient numbers of this species and *An. triannulatus* precluded some analyses conducted on the more abundant species.

Several species showed pre-dawn increases in host seeking during the final hour of nightly collections (e g, of *An. darlingi*) (Figure 9, Additional file 1), *An. marajoara* (Figure 4, Additional file 1) and *An. triannulatus* (Figure 8, Additional file 4). Possibly these same species might also have been captured after dawn, if collections had been made, which would further alter skewness and kurtosis moments and interpretations.

Charlwood and others [3] hypothesized that biting patterns may have evolved in relation to host choice; with anthropophilic species being nocturnal and zoophilic species being crepuscular. Bloodmeal host identifications in the study area [22] indicated that the most crepuscular species, *An. nuneztovari* and *An. triannulatus* were more zoophilic compared to the more nocturnally active *An. darlingi* and *An. marajoara*. These results seem to support a host choice hypothesis. However, the authors formerly showed that host availability was the most important factor in determining host blood meal choice in these same three villages [22].

### Inferences for malaria control

The degree of kurtosis indicates whether the risk of being bitten occurs over a short time period (leptokurtic), is normally distributed (mesokurtic), or occurs over a longer period (platykurtic; Figure 1). In this study *An. nuneztovari* and *An. triannulatus*, were mainly crepuscular being most active from 17:30 to 21:30. They both frequently showed positive skewness and high leptokurtosis. Therefore, vector control programmes would need to consider developing a control component that includes the time period when people are still active outside. *Anopheles darlingi* was active at night (17:30–06:30), had mainly non-significant skewness and non-significant kurtosis (mesokurtic). In this case, assuming that host-seeking activity patterns are similar for exophagic and endophagic *An. darlingi*, an indoor vector control strategy, such as impregnated materials [28-30] may work the best. It is more difficult to determine a risk activity period for species like *An. marajoara* and *An. intermedius*. *Anopheles marajoara* had positive skewness but a lower frequency of positive kurtosis than did *An. nuneztovari* and *An. triannulatus*. Its activity pattern was more dispersed with additional activity throughout the night. *Anopheles intermedius* had no striking difference in the percentage of significantly positive skewness and kurtosis compared to non-significant distributions (Table 5 and Table 8). One would have to take into account other risk factors related to malaria transmission before recommending vector control for these two species.

The appropriate methods of malaria and vector control are highly dependent on the epidemiology of malaria in a particular location, including Plasmodium species, vector competency, health service availability and control methods [5,7]. In the study area the presence of five potential vectors [21] with different biting cycles further complicates any vector management programme. Previous research in these villages showed that *An. darlingi* and *An. marajoara* are the most abundant and anthropophilic species [22,23] and have the highest *Plasmodium* spp. infection rates [21]. Therefore, one would most likely focus on these two species when developing an integrated malaria control programme for this region of the Brazilian Amazon.

## Conclusion

The use of the co-efficient of variation, skewness and kurtosis, to dissect and compare the nightly biting of anophelines provided statistical tools for analysing host-seeking activity patterns. It clarified ambiguous differences in distribution shape that were observed between species, sites, villages and dates. Variation in the biting cycle of anophelines impacts malaria control programmes. Results of this study help fill gaps in the knowledge about the biting behaviour of anophelines in the Amazon Basin [30].

## Competing interests

The authors declare that they have no competing interests.

## Authors’ contributions

LPL helped design the study, analysed the results, and drafted the manuscript. RHZ helped design the study, carried out field studies, and drafted the manuscript. NN organized and analysed data. AKRG conducted fieldwork, organized field operations, identified the anophelines collected during the study, and participated in the review of the data. CG and MEA were involved in field studies and the laboratory analysis of data. All authors have read and approved the final manuscript.

## Supplementary Material

Additional file 1 **Mean (±SE) monthly human landing catch (HLC) for *An. darlingi*,*An. marajoara *and *An. nuneztovari *from April 2003 to November 2003 in Santo Antônio.** Click here for file

Additional file 2 **Mean (±SE) monthly biting activity for *An. darlingi*, *An. marajoara *and *An. nuneztovari *in São Raimundo.** Click here for file

Additional file 3 **Mean monthly human landing catch for *An. darlingi*, *An. marajoara *and *An. nuneztovari *from April 2003 to October 2005 in São João.** Click here for file

Additional file 4 **Mean monthly human landing catch for *An. intermedius *and *An. triannulatus *from April 2003 to November 2005.** Click here for file

Additional file 5 **Results of nested ANOVAs to detect sources of variation in skewness and kurtosis of *An. darlingi *and *An. marajoara *in three villages.** Click here for file

## References

[B1] Elliot R Studies on man-vector contact in some malarious areas in Colombia Bull World Health Organ 1968 38 239 253 5302300 PMC2554320

[B2] Elliot R The influence of vector behavior on malaria transmission AmJTrop Med Hyg 1972 21 755 763 10.4269/ajtmh.1972.21.755 4561523

[B3] Charlwood JD Biological variation in *Anopheles darlingi* Root Mem Inst Oswaldo Cruz 1996 91 391 398 9070397 10.1590/s0074-02761996000400001

[B4] Zimmerman RH Voorham J Use of insecticide-impregnated mosquito nets and other impregnated materials for malaria control in the Americas Rev Panam Salud Publica 1997 2 18 25 10.1590/S1020-49891997000700004 9309945

[B5] Giglioli G Biological variations in *Anopheles darlingi* and *Anopheles gambiae*. Their effect on practical malaria control in the neotropical region Bull World Health Organ 1956 15 461 471 13404433 PMC2538274

[B6] Gabaldón A Difficulties confronting malaria eradication AmJTrop Med Hyg 1972 21 634 639 10.4269/ajtmh.1972.21.634 5074686

[B7] Zimmerman RH Braga I (Eds): Controle Seletivo de Vectores da 1999 Guia para o Nivel Municipal. Fundacão Nacional de Saúde/Organizacão Pan-Americana de Saúde: Malária

[B8] Zimmerman RH The ecology of malaria vectors in the Americas and future direction Mem Inst Oswaldo Cruz 1992 87 371 383 1343717 10.1590/s0074-02761992000700064

[B9] Hudson JE *Anopheles darlingi* Root (Diptera: Culicidae) in the Suriname rain forest Bull Entomol Res 1984 74 129 142 10.1017/S0007485300010002

[B10] Rubio-Palis Y Observaciones sobre el patrón de actividad hematofágica del vector de malaria *Anopheles darlingi* en poblaciones del sur de Venezuela Bol Dir Malariol San Amb 1995 35 66 70

[B11] Magris M Rubio-Palis Y Menares C Villegas L Vector bionomics and malaria transmission in the Upper Orinoco River, Southern Venezuela Mem Inst Oswaldo Cruz 2007 102 303 311 10.1590/S0074-02762007005000049 17568935

[B12] Leon C Valle J Naupay R Tineo E Rosas A Palomino R Comportamiento estacional del *Anopheles* (*Nyssorhynchus*) *darlingi* Root 1926 en localidades de Loreto y Madre de Dios, Perú 1999–2000 Rev Peruana Med Exper Sal Pub 2003 20 22 27

[B13] Pajot F Le Pont F Molez J Degallier N Agressivité d’*Anopheles* (*Nyssorhynchus*) *darlingi* Root, 1926 (Diptera: Culicidae) en Guyane Française Cahiers ORSTOM ser Ent med et Parasit 1977 15 15 22

[B14] Forattini OP Comportamento exofilo de *Anopheles darlingi* Root, em regiao meridional do Brasil Rev Saude Pub Sao Paulo 1987 21 291 304 10.1590/s0034-89101987000400002 3445112

[B15] Roberts DR Alecrim WD Tavares AM Radke MG The house-frequenting, host-seeking and resting behavior of *Anopheles darlingi* in southeastern Amazonas, Brazil J Am Mosq Control Assoc 1987 3 433 441 3504928

[B16] Voorham J Intra-population plasticity of *Anopheles darlingi*’s (Diptera, Culicidae) biting activity patterns in the state of Amapá Brazil Rev Saude Pub 2002 36 75 80 10.1590/S0034-89102002000100012 11887233

[B17] Deane M Causey OR Deane MP Notas sobre distribuição e a biologia dos anofelinos das Regioes Nordestina e Amazônica do Brasil Rev Serv Esp Saude Pub 1948 1 827 965

[B18] Rubio-Palis Y Curtis CF Biting and resting behaviour of anophelines in western Venezuela and implications for control of malaria transmission Med Vet Entomol 1992 6 325 334 10.1111/j.1365-2915.1992.tb00628.x 1463897

[B19] Sokal RR Rohlf FJ Biometry: the Principles and Practice of Statistics in Biological Research 2012 4 New York: W. H. Freeman and Co

[B20] DeCarlo LT On the meaning and use of kurtosis Psychol Meth 1997 2 292 307

[B21] Galardo AKR Arruda M Couto AR Wirtz R Lounibos LP Zimmerman RH Malaria vector incrimination in three rural riverine villages in the Brazilian Amazon AmJTrop Med Hyg 2007 76 461 469 17360868

[B22] Zimmerman RH Galardo AKR Lounibos LP Arruda M Wirtz RA Bloodmeal hosts of *Anopheles* species (Diptera: Culicidae) in a malaria endemic area of the Brazilian Amazon J Med Entomol 2006 43 947 956 10.1603/0022-2585(2006)43[947:BHOASD]2.0.CO;2 17017232

[B23] Galardo AKR Zimmerman RH Lounibos LP Young LJ Galardo D Arruda M D’Almeida Couto AR Seasonal abundance of anopheline mosquitoes and their association with rainfall and malaria along the Matapí River, Amapá, Brazil Med Vet Entomol 2009 23 335 349 10.1111/j.1365-2915.2009.00839.x 19941599

[B24] Consoli RAGB Lourenço de Oliveira R: Principais Mosquitos de Importância Sanitária no Brasil 1994 Rio de Janeiro: Fiocruz

[B25] SAS Institute Inc: SAS/STAT® 9.2 User’s Guide SAS Institute Inc 2008 NC: Cary

[B26] Charlwood JD Hayes J Variações geográficas no ciclo de picada do *Anopheles darlingi* Root no Brasil Acta Amazoníca 1978 8 605 61123838179

[B27] Conn JE Wilkerson RC Segura MNO Souza RTL Schlichting CD Wirtz RA Povoa MM Emergence of a new Neotropical malaria vector facilitated by human migration and changes in land use AmJTrop Med Hyg 2002 66 18 22 10.4269/ajtmh.2002.66.18 12135261

[B28] Voorham J The use of wide-mesh gauze impregnated with lambda-cyhalothrin covering wall openings in huts as a vector control method in Suriname Rev Saude Pub 1997 31 9 14 10.1590/S0034-89101997000100003 9430921

[B29] Hiwat H Mitro S Samjhawan A Sardjoe P Soekhoe T Takken W Collapse of *Anopheles darlingi* populations in Suriname after introduction of insecticide-treated nets (ITNs); malaria down to near elimination level AmJTrop Med Hyg 2012 86 649 655 10.4269/ajtmh.2012.11-0414 PMC3403763 22492150

[B30] Da Silva-Nunes M Moreno M Conn JE Gamboa D Abeles S Vinetz JM Ferreira MU Amazonian malaria: Asymptomatic human reservoirs, diagnostic challenges, environmentally driven changes in mosquito vector populations, and the mandate for sustainable control strategies Acta Trop 2012 121 281 291 10.1016/j.actatropica.2011.10.001 22015425 PMC3308722

