# Supplementary material for: Nightly biting cycles of malaria vectors in a heterogeneous transmission area of eastern Amazonian Brazil
Source: Malar J. 2013 Jul 26;12:262. doi: 10.1186/1475-2875-12-262 (PMC3729824; doi:10.1186/1475-2875-12-262)

Additional file 1 Mean (±SE) monthly human landing catch (HLC) for *An. darlingi*, *An. marajoara* and *An. nuneztovari* from April 2003 to November 2003 in Santo Antônio

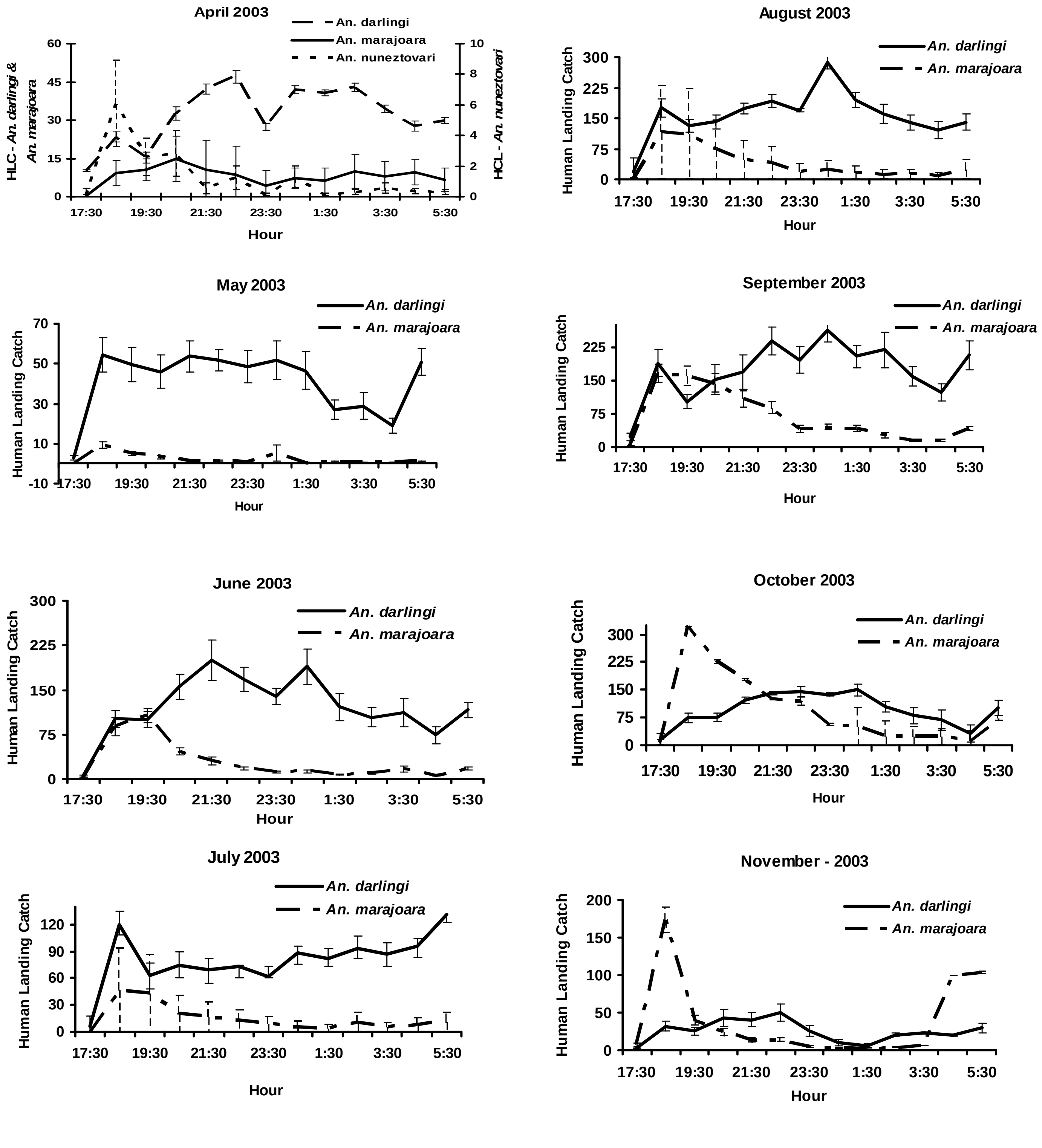

Additional file 1 (cont) Mean (±SE) monthly human landing catch (HLC) for *An. darlingi*, *An. marajoara* and *An. nuneztovari* from February 2004 to September 2004 in Santo Antônio

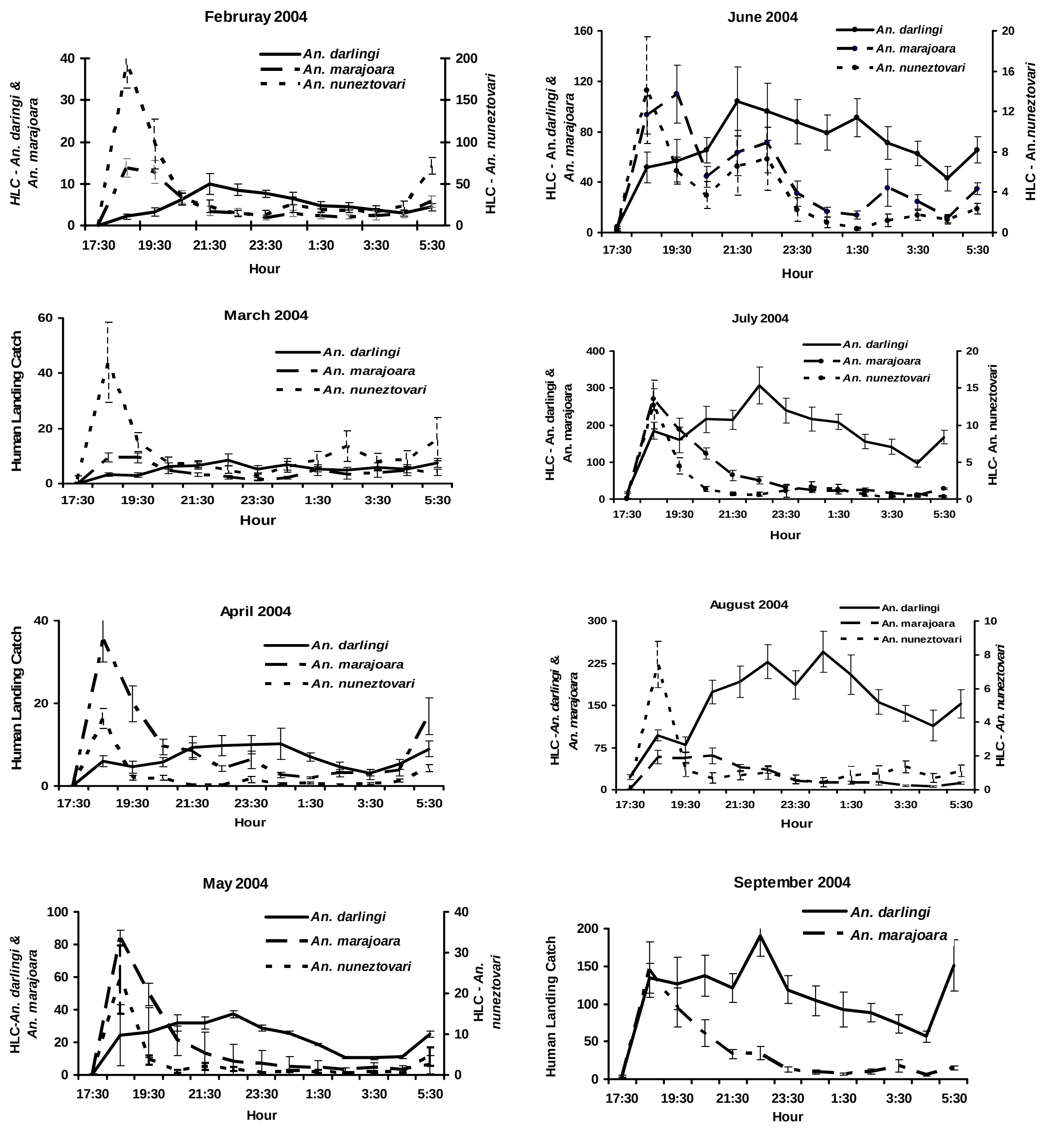

Additional file 1 (cont) Mean (±SE) monthly human landing catch (HLC) for *An. darlingi*, *An. marajoara* and *An. nuneztovari*, October 2004 and November 2004 in Santo Antônio

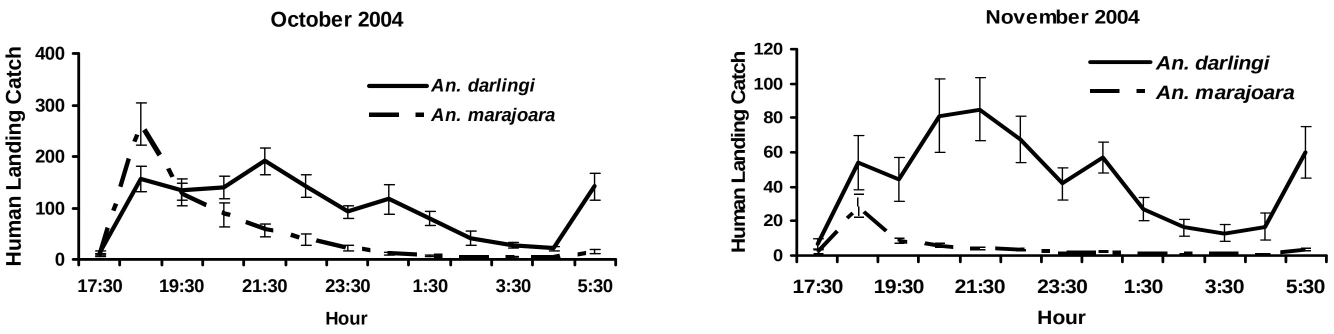

Additional file 1 (cont) Mean (±SE) monthly human landing catch (HLC) for *An. darlingi*, *An. marajoara* and *An. nuneztovari* from February 2005 to September 2005 in Santo Antônio

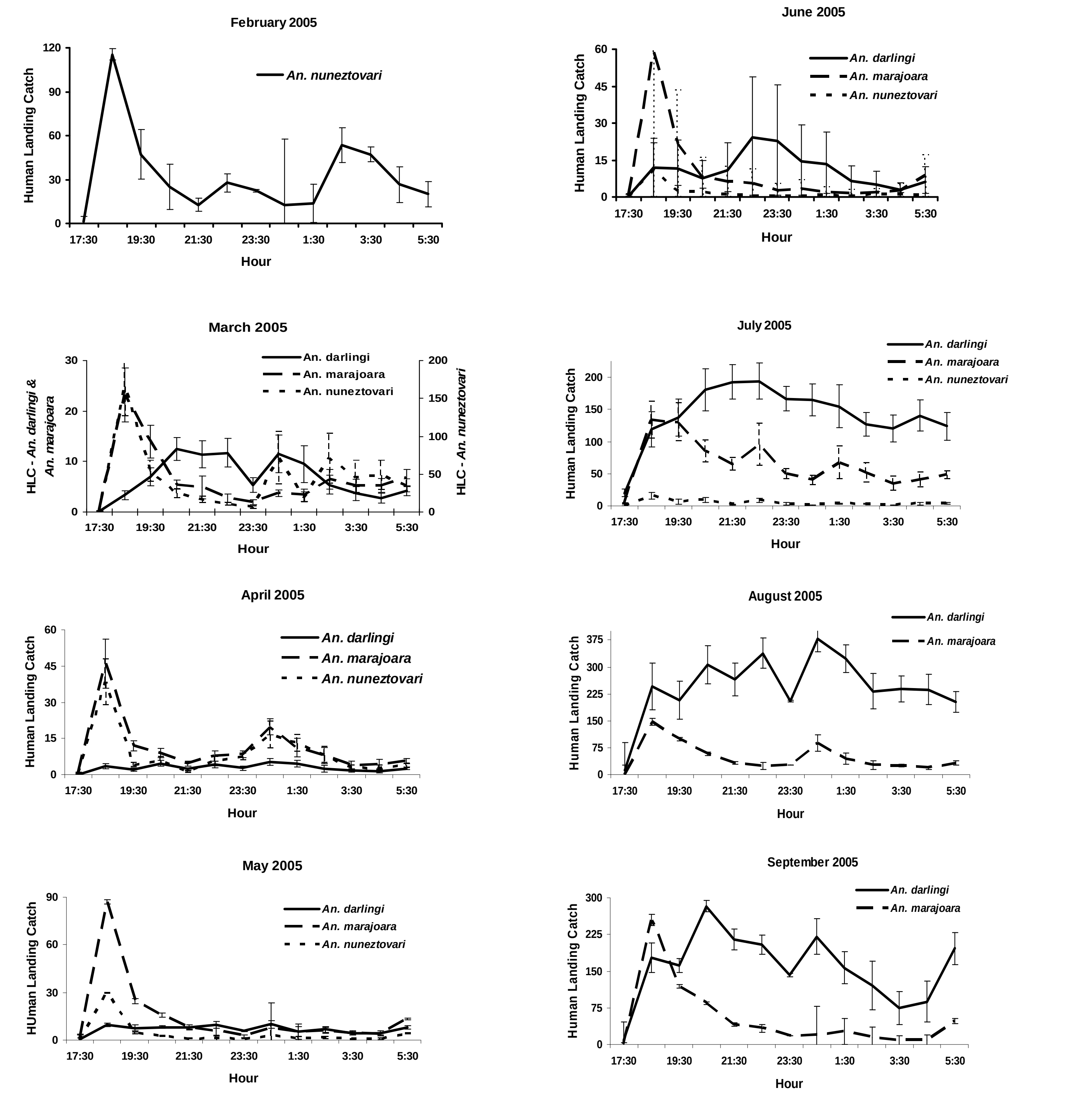

Additional file 1 (cont.) Mean (±SE) monthly human landing catch (HLC) for *An. darlingi, An. marajoara* and *An. nuneztovari*, October 2005 and November 2005 in Santo Antônio

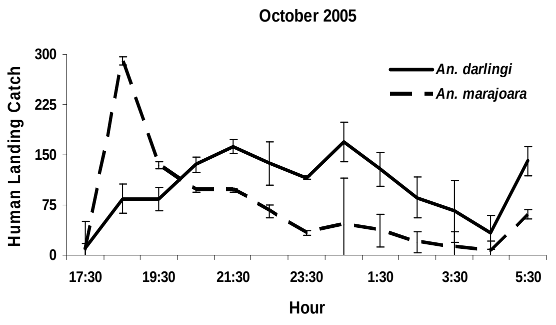

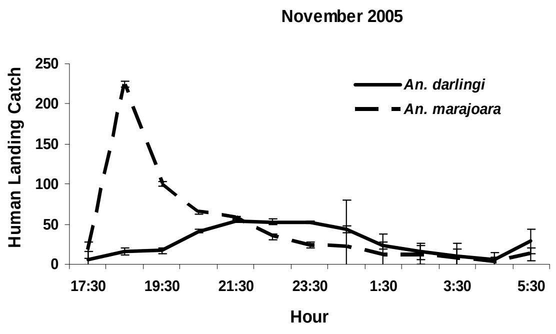

Supplement: Additional file 1 — Mean (±SE) monthly human landing catch (HLC) for An. darlingi,An. marajoara and An. nuneztovari from April 2003 to November 2003 in Santo Antônio. [file 1475-2875-12-262-S1.pdf]
