# Supplementary material for: Nightly biting cycles of malaria vectors in a heterogeneous transmission area of eastern Amazonian Brazil
Source: Malar J. 2013 Jul 26;12:262. doi: 10.1186/1475-2875-12-262 (PMC3729824; doi:10.1186/1475-2875-12-262)

Additional file 2 Mean (±SE) monthly human landing catch for *An. darlingi, An. marajoara* and *An. nuneztovari* in São Raimundo

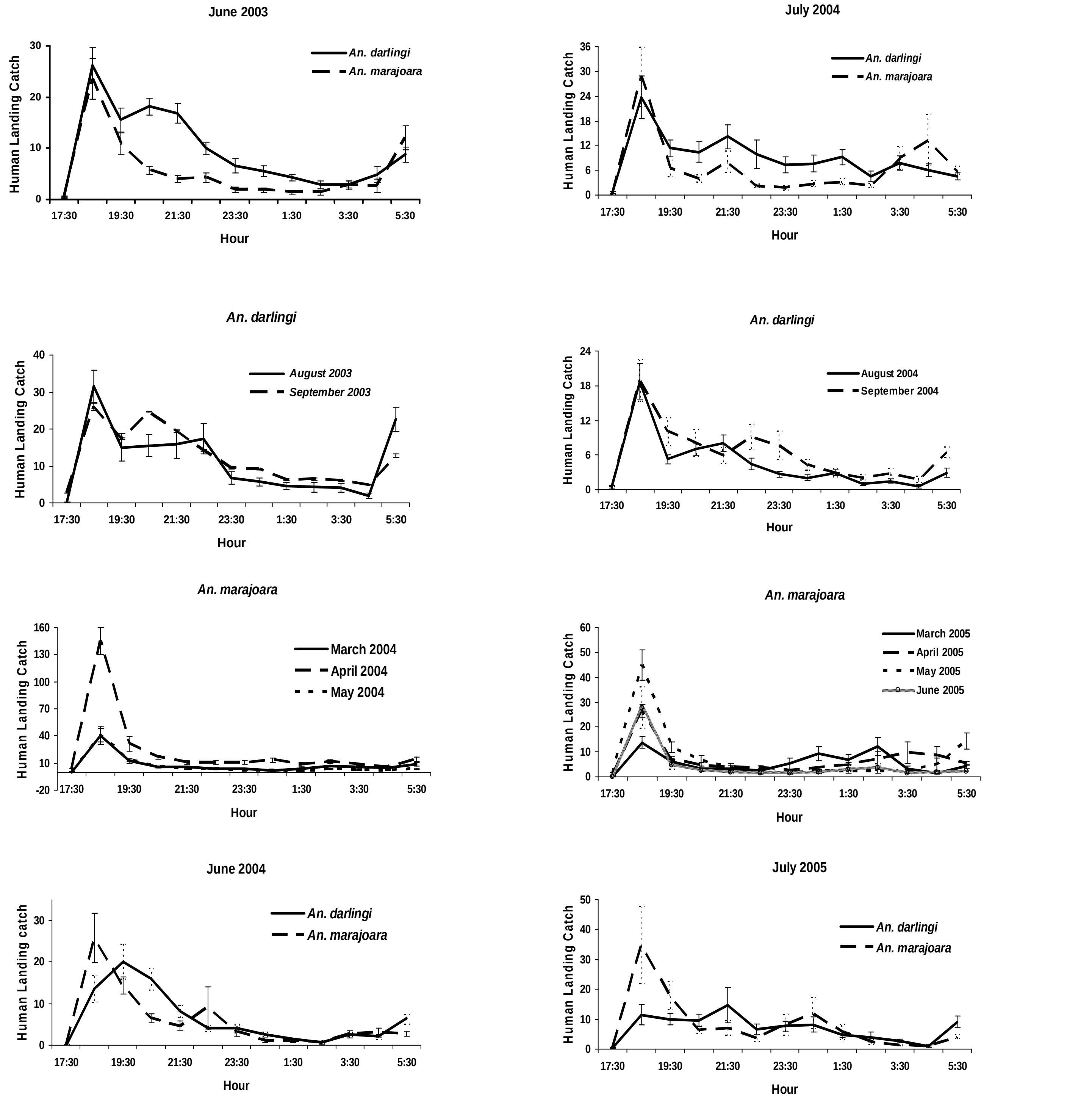

Additional file 2 (cont) Mean (±SE) monthly human landing catch for *An. darlingi, An. marajoara* and *An. nuneztovari* in São Raimundo

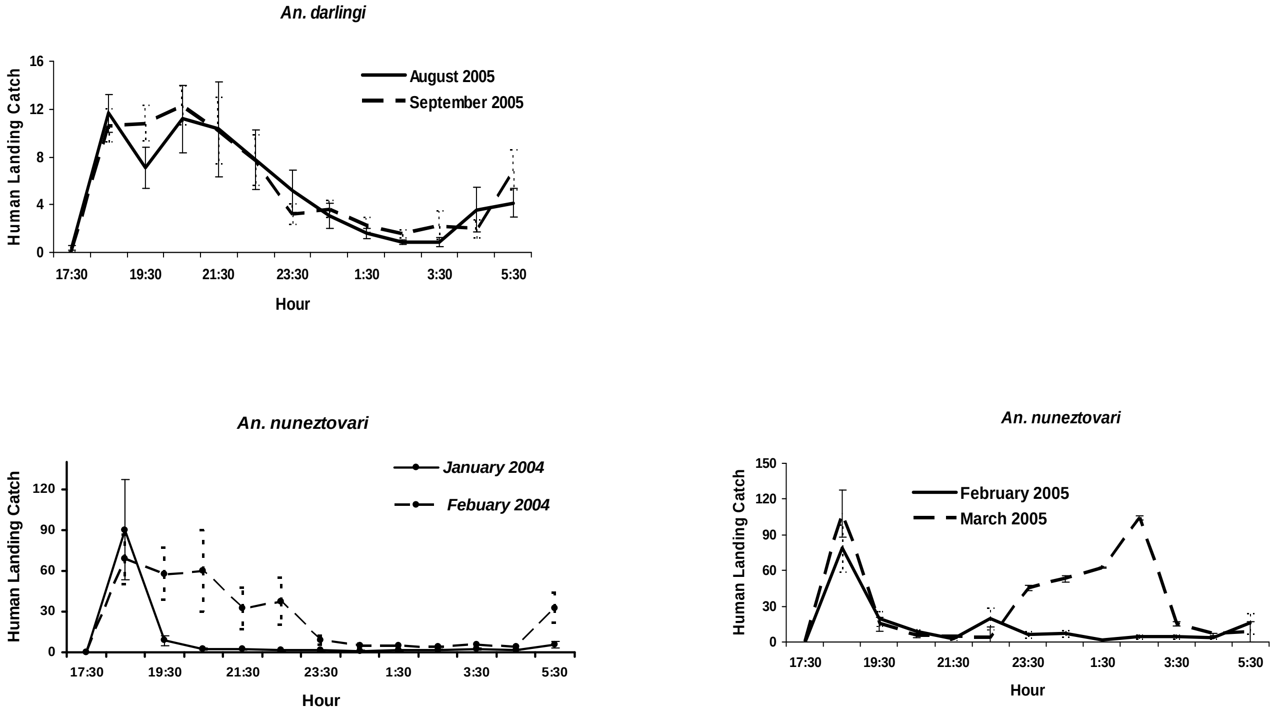

Supplement: Additional file 2 — Mean (±SE) monthly biting activity for An. darlingi, An. marajoara and An. nuneztovari in São Raimundo. [file 1475-2875-12-262-S2.pdf]
