# Supplementary material for: Nightly biting cycles of malaria vectors in a heterogeneous transmission area of eastern Amazonian Brazil
Source: Malar J. 2013 Jul 26;12:262. doi: 10.1186/1475-2875-12-262 (PMC3729824; doi:10.1186/1475-2875-12-262)

Additional file 4 Mean monthly human landing catch for *An. intermedius* and *An. triannulatus*

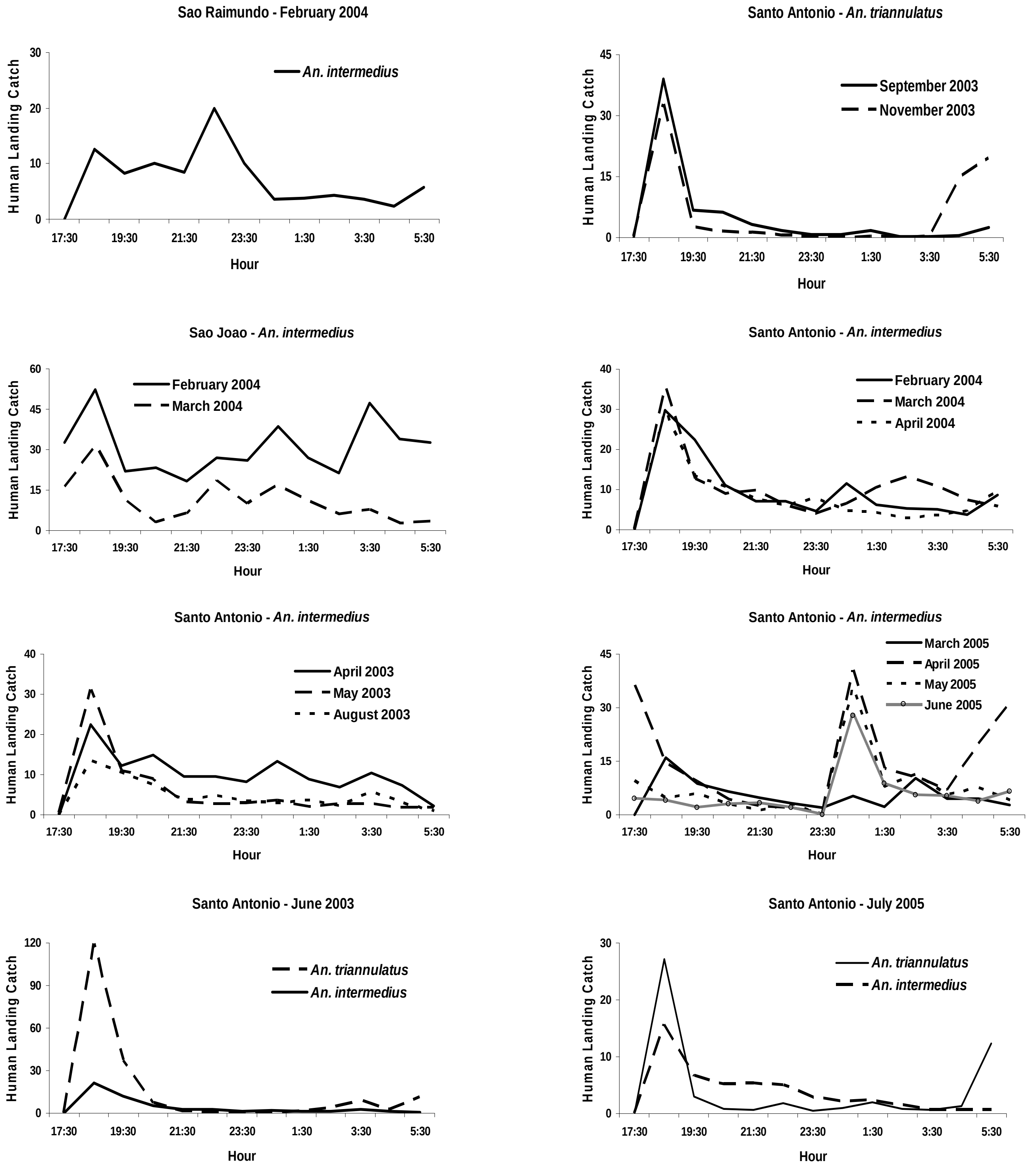

Supplement: Additional file 4 — Mean monthly human landing catch for An. intermedius and An. triannulatus from April 2003 to November 2005. [file 1475-2875-12-262-S4.pdf]
