# Supplementary material for: Nightly biting cycles of malaria vectors in a heterogeneous transmission area of eastern Amazonian Brazil
Source: Malar J. 2013 Jul 26;12:262. doi: 10.1186/1475-2875-12-262 (PMC3729824; doi:10.1186/1475-2875-12-262)
Supplement: Additional file 5 — Results of nested ANOVAs to detect sources of variation in skewness and kurtosis of An. darlingi and An. marajoara in three villages. [file 1475-2875-12-262-S5.pdf]

Additional file 5 Results of nested ANOVAs to detect sources of variation in skewness and kurtosis of *An. darlingi* and *An. marajoara* in three villages

---

Table 1 Results of nested ANOVA for skewness (g_1) of *An. darlingi* hourly landing rates in three Amazonian villages

	Model				Year		Month	(year)	night	(yearXmo.)	site	(yearXmo.)
village	d.f.	F	R^2	mean g_1	d.f.	F	d.f.	F	d.f.	F	d.f.	F
SA	163	1.04ns	0.523	0.585	2	1.77ns	24	1.30ns	52	0.91ns	84	1.01ns
SJ	124	1.02ns	0.573	1.07	2	0.25ns	20	1.71*	37	0.94ns	62	0.79ns
SR	84	13.79***	0.955	1.93	2	10.28**	15	22.75***	25	29.01***	41	1.13ns

Table 2 Results of nested ANOVA for skewness (g_1) of *An. marajoara* hourly landing rates in three Amazonian villages

	Model				Year		Month	(year)	night	(yearXmo.)	site	(yearXmo.)
village	d.f.	F	R^2	mean g_1	d.f.	F	d.f.	F	d.f.	F	d.f.	F
SA	161	2.66***	0.747	1.84	2	0.51ns	26	6.04***	51	2.05***	81	1.57**
SJ	137	2.81***	0.852	2.29	2	2.73ns	22	6.11***	44	2.00**	69	1.48ns
SR	74	4.91***	0.894	0.511	2	2.46ns	11	8.59***	22	7.44***	38	1.65ns

Table 3 Results of nested ANOVA for kurtosis (g_2) of *An. darlingi* hourly landing rates in three Amazonian villages

	Model				Year		Month	(year)	night	(yearXmo.)	site	(yearXmo.)
village	d.f.	F	R^2	mean g_2	d.f.	F	d.f.	F	d.f.	F	d.f.	F
SA	163	1.27ns	0.571	0.503	2	2.99ns	24	1.10ns	52	1.47*	84	1.19ns
SJ	124	0.92ns	0.547	1.19	2	0.43ns	20	1.50ns	37	1.02ns	62	0.59ns
SR	84	4.06***	0.863	2.48	2	2.15ns	15	7.73***	25	4.59***	41	2.59**

Table 4 Results of nested ANOVA for kurtosis (g_2) of *An. marajoara* hourly landing rates in three Amazonian villages

	**Model**				**Year**		**Month**	**(year)**	**night**	**(yearXmo.)**	**site**	**(yearXmo.)**
**village**	**d.f.**	**F**	R^2	**mean g_2**	**d.f.**	**F**	**d.f.**	**F**	**d.f.**	**F**	**d.f.**	**F**
SA	161	2.38***	0.725	3.84	2	0.04ns	26	5.96***	51	1.56*	81	1.44**
SJ	137	2.78***	0.850	6.12	2	2.40ns	22	5.88***	44	2.24***	69	1.48ns
SR	74	5.23***	0.900	6.48	2	2.90ns	11	9.73***	22	7.79***	38	1.66ns
